# A comprehensive guava leaves and fruits dataset for guava disease recognition

**DOI:** 10.1016/j.dib.2022.108174

**Published:** 2022-04-12

**Authors:** Aditya Rajbongshi, Sadia Sazzad, Rashiduzzaman Shakil, Bonna Akter, Umme Sara

**Affiliations:** a Department of Computer Science and Engineering, National Institute of Textile Engineering and Research, Dhaka, Bangladesh; b Department of Computer Science and Engineering, Daffodil International University, Dhaka, Bangladesh

**Keywords:** Guava disease, Recognition, Machine learning, Deep learning

## Abstract

Guava (Psidium guajava) is a delicious fruit native to Mexico, Central or South America, and the Caribbean region. It's high in vitamin C, Calcium, Pectins and is a good source of fiber. Due to concerns with natural and environmental resources, technical issues, and other impediments, the production level decreases day-to-day. However, we'll concentrate on the most critical challenges, such as infections that affect guava plants, fruits, and disease outbreak prevention through early identification. Besides, the early recognition of guava disease using the expert system will lead to higher yields that will eventually help guava farmers reduce their economic losses. In the recent era, image processing and computer vision have been broadly applied to recognize multiple diseases that are not identified with the naked eyes. This article presents a dataset of guava images containing both leaves and fruit images (diseases affected and disease-free) are classified into six classes: for guava fruits-Phytophthora, Scab, Styler end Rot, and Disease-free fruit, and for guava leaves-Red Rust, and diseases-free leave. All images are basically captured from the guava garden located at Bangladesh Agricultural University in July when the guava fruits are almost ripened, and the infections are found in guava plants. This dataset is mainly for those researchers who work with computer vision, machine learning, and deep learning to develop a system that recognizes the guava disease to assist guava farmers in their cultivation.

## Specifications Table

**Table:** 

Subject	Computer Science
Specific subject area	Image processing, Image detection, Image classification, and computer vision [1]
Type of data	Images
How the data were acquired	Firstly, the guava garden is selected by analyzing the area where those gardens are mostly situated in Bangladesh. Among several places, the guava garden has been selected by an expert in this section, which has a large scale of guava production and a variety of disease-affected plants and fruits in the middle of the year 2021 (July). Finally, after visiting that place with the recommendation of the expertise, the images are collected using a Digital SLR Camera.
Data format	Raw
Description of data collection	There was no such sample pre-treatment. With the help of a domain expert and a plant research centre, the images have been accumulated manually from the guava garden.
Data source location	**Institution:** Bangladesh Agriculture University **City:** Mymensingh, Dhaka **Country:** Bangladesh
Data accessibility	**Repository name:** Mendeley Data **Data identification number (permanent identifier, i.e., DOI number):**10.17632/x84p2g3k6z.2 **Direct link to the dataset:**https://data.mendeley.com/datasets/x84p2g3k6z/1

## Value of the Data

• This dataset when used in machine learning and deep learning models helps early detection and classification of guava diseases which is essential to developing a well-performed automated agricultural system.
• Researchers can utilize this data to create an automated system that farmers may use to boost production and contribute to the global economy significantly.
• The dataset of guava diseases can be utilized to segment, detect, and classify early signs of diseases which can be found in Guava plants and fruits, through various machine learning and deep learning models. As the dataset has been collected from the field level, the researchers will get a noteworthy outcome by utilizing machine learning and deep learning models.
• Early detection of guava diseases can assist farmers in producing higher yields of products, resulting in improvement of their socioeconomic stability.
• Early detection of guava disease is required for widespread guava production; as a consequence, the dataset will assist the researcher and farmer in overcoming numerous challenges.

## 1 Data Description

Guava is very nutritious and one of the sweetest fruits in the world which is high in fiber and a good source of essential vitamins and minerals [2]. Besides, it contains a variety of health-promoting antioxidants, including flavonoids. However, guava production is decreasing these days due to several diseases that farmers cannot notice with their naked eyes. As they are not properly trained and educated, they cannot consult with agricultural specialists at the right time.

Approximately 177 pathogens have been found in abundant portions of the guava plant or connected with guava [3]. Aside from that, fruit and post-harvest infections are significant in causing numerous losses. The four typical diseases that are found in guava plants and fruits are emphasized in this article. Besides, the disease-free images are also comprised for the proper classification in machine learning and deep learning models. The detailed description of the utilized dataset is presented in Table 1.

**Table 1:** Brief description of the dataset.

Disease Name	Description	Visualization
Phytopthora	One of the most frequent guava diseases is Phytopthora, which is caused by fungus and manifests as black lesions on young fruits. Because of the expansion of fruits, lesions become shallow and fractured more commonly on older imperfections. Lesions are generally smaller than leaf marks. They range in hue from dark green to brown to black.	
Red rust	Red rust is also caused by a fungus that causes circular lesions that develop orange to red pustules on leaves, young shoots, deformed leaves, black borders, and yellow halos. There is no thallus over the surface of the leaf, the lesions proliferate alongside the entire lamina, and sporulation happens upon that disease exterior on the leaf's surface. The primary cause of this condition is excessive humidity and warm temperatures.	
Scab	Scab is caused by fungi bearing the symptoms of ovoid, corky, and round lesions on the surface of guava fruits. These lesions are affecting the flesh beneath, diminishing fruit quality and production value. Unripe fruits are mainly affected by fungus, which creates dark and brown scabby lesions measuring 2-4mm in diameter. The peel of the fruit beneath the white cottony maturation process turns soft and embellishes light brown to tragic brown. Fruits at the soil level that are covered with thick foliage are adversely damaged by high relative humidity. The falling fruits have been severely harmed.	
Styler end rot	The disease begins at the styler and spreads to the root in guava fruits. The white cottony growth usually appears when the fruit matures and can envelop the whole surface in a period of 3-4 days throughout humid climates. Fruits at the humus surface coated with opaque foliage and exposed to higher humidity appear to be the most seriously damaged. The epidermis of the fruit behind the whitish cottony development of mycelium softens, becomes brownish color to dark brown, and produces a distinctly disagreeable odor. When the illness affects immature or partially grown fruits, they wither, become dirty brown to dark brown, remain rigid in structure, and stay whole as mummified fruit or fall off.	
Disease-free (leave)	The fruits are annular to pear-shaped with an estimation of 7.6 cm in diameter. The flesh of the guava fruit contains countless little solid seeds. The flesh is white, yellow, or pink, with yellow skin. Besides the leaves are approximately 7-15cm long and 3-5cm wide having different shades of green color. When crushed, guava leaves emit a perfume that is comparable to that of the guava fruit [4]. Guava leaves are found on a tiny tree with extensive branches and peeling copper-colored bark that reveals a green base.	
Diseases-free (fruit)	Guava fruits range in size from 4 to 12 centimeters in length, depending on the species. They have a distinct and distinctive scent that is akin to lemon peel but less harsh. The outer skin might be harsh and bitter, or it can be delicate and pleasant. The skin can be any thickness and is normally green before maturity, but can be yellow, maroon, or green when mature, depending on the species. The pulp inside can be sweet or sour and ranges in color from off-white (“white”) to deep pink (“red” guavas) the amount and hardness of the seeds in the center pulp vary.	

This article presents the dataset of guava (disease affected, and disease-free) containing a total of 681 images that are acquired from the guava garden of Bangladesh Agriculture University, Mymensingh, Dhaka. As the diseases of guava plants are mostly found in guava fruits and leaves, the dataset contains both fruits and leaves sample images for diseases recognition. The images of guava leaves and fruits are collected manually by the Digital SLR Camera with the help of experts and researchers in this domain. Then the composed original images are augmented using image processing techniques. All the images are resized with a generalized dimension of 512 × 512. Table 2 depicts the distribution of those images. This dataset is valid for the researchers who work with machine learning and deep learning models in agriculture, especially for guava disease recognition.

**Table 2:** Class wise dataset distribution.

Class Name	Amount of Original Data	Amount of Augmented Data
		
Phytopthora	114	1342
Red Rust	87	1554
Scab	106	1264
Styler end Rot	94	1463
Disease-free (leave)	126	1276
Disease-free (fruit)	154	1626
Total	681	8525
		

## 2 Experimental Design, Materials and Methods

### 2.1 Camera specification

The dataset was acquired using an advanced Nikon Digital SLR Camera D3200 with a Nikon F mount with an effective angle of view having approximately 1.5x lens focal length. This digital SLR camera has a CMOS sensor and a resolution of 23.2 × 15.4 mm. The chosen frame advance rate is 4fps manual focus, shutter speed 1/250 s, and other settings at default values.

### 2.2 Prepossessing

**Fig. 1:**
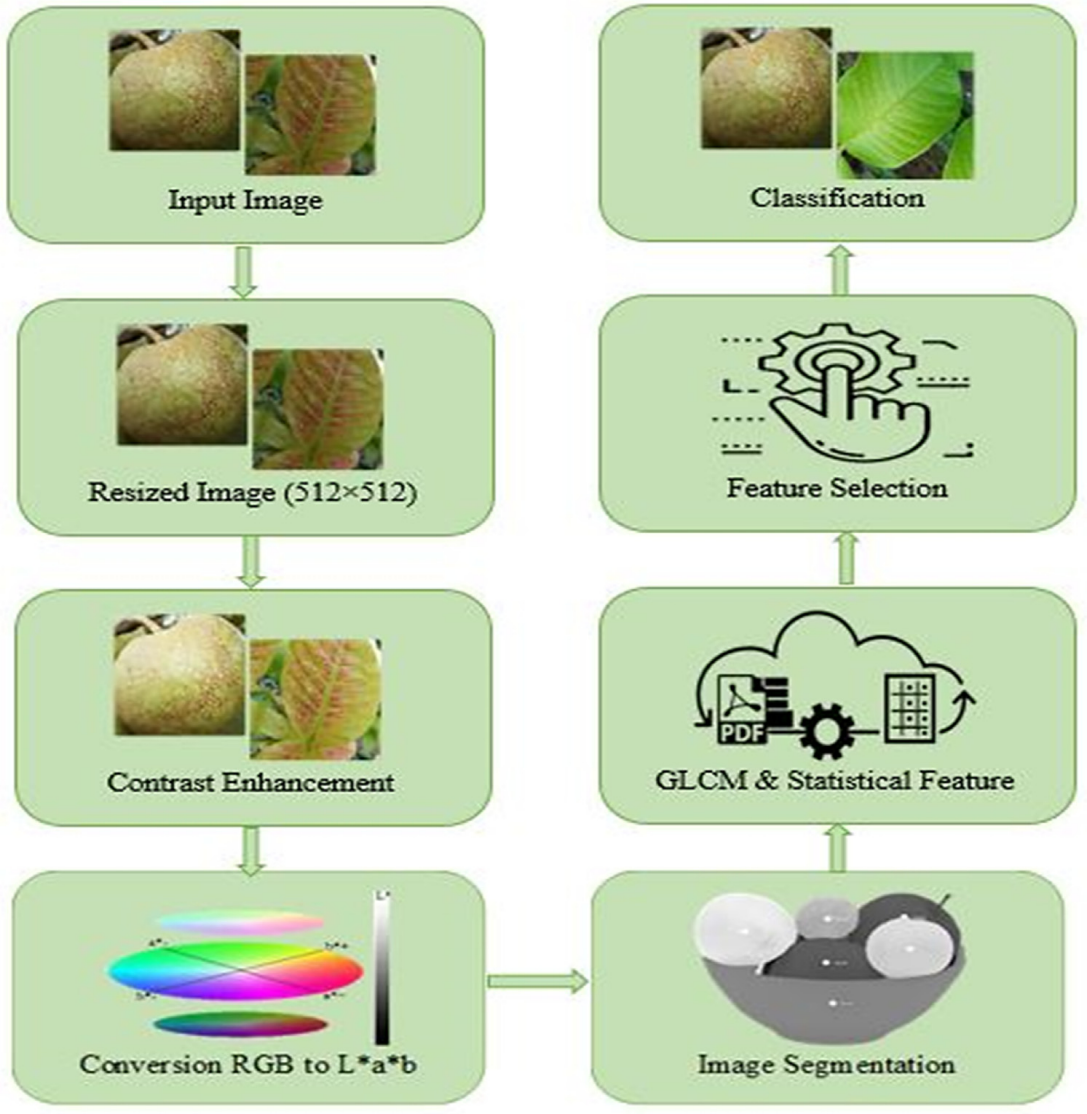
Processing steps of guava disease classification.

The collected images are different in size and shape as those are captured randomly from the guava garden. For starting the process, all the collected images were resized into a pedestrian size of 512 × 512 using the Bicubic interpolation. Each and every image that entered through the system was combined with six steps such as (i) contrast enhancement, (ii) color space conversion, (iii) segmentation, (iv) feature extraction, (v) feature selection, and (vi) classification that is also described in Fig. 1. The contrast of each image is enhanced by applying the histogram equalization technique. Then color conversion is performed to achieve the L*a*b color space from RGB color space as all colors are feasible in L*a*b color space, and segmentation operation is performed well. After that, the segmentation operation (K-means clustering) is applied to isolate the disease-affected region. Table 3 shows the segmentation procedure of guava images. After segmentation, two features, Gray Level Co-occurrence Matrix (GLCM) and Statistical features are extracted [5]. All of the GLCM and Statistical features are not effective to classify guava diseases. Some features affect the most which is why feature selection plays a significant role in guava disease classification. Furthermore, the property specification is performed using various feature selection methods and then those features are utilized to train and test the applied classifiers. Finally, guava disease classification is accomplished for different classifiers of machine learning.

**Table 3:** Segmentation procedure for the original image.

## Ethical Approval (Involvement of Animals)

This article does not contain any studies with animals performed by any of the authors.

## Ethical Approval (Involvement of Human Subjects)

There are no studies involving human participants done by any of the authors in this article. The datasets used in the article are open to the public. For the usage of these datasets, proper citation rules should be maintained.

## CRediT authorship contribution statement

**Aditya Rajbongshi:** Conceptualization, Methodology, Software. **Sadia Sazzad:** Data curation, Visualization, Writing – original draft. **Rashiduzzaman Shakil:** Software, Validation. **Bonna Akter:** Writing – review & editing. **Umme Sara:** Supervision, Investigation.

## Declaration of Competing Interest

The authors declare that they have no known competing financial interests or personal relationships that could have appeared to influence the work reported in this paper.

## Data Availability

Guava Leaves and Fruits Dataset for Guava Disease Recognition through Machine Learning and Deep Learning (Original data) (Mendeley Data).
